# Efficacy of Automatic 3D Segmentation of the Upper Airway in CBCT or CT Scans via Artificial Intelligence Versus Manual Segmentation by Human Experts: A Systematic Review and Meta‐Analysis

**DOI:** 10.1002/cre2.70314

**Published:** 2026-03-01

**Authors:** Farhad Sobouti, Mehdi Aryana, Hossein Mohammad‐Rahimi, Sepideh Dadgar, Reza Alizadeh‐Navaei, Vahid Rakhshan

**Affiliations:** ^1^ Dental Research Center Mazandaran University of Medical Sciences Sari Iran; ^2^ Orthodontic Department, Faculty of Dentistry Mazandaran University of Medical Sciences Sari Iran; ^3^ Faculty of Dentistry Mazandaran University of Medical Sciences Sari Iran; ^4^ Topic Group Dental Diagnostics and Digital Dentistry, ITU/WHO Focus Group AI on Health Berlin Germany; ^5^ Gastrointestinal Cancer Research Center, Noncommunicable Diseases Institute Mazandaran University of Medical Sciences Sari Iran; ^6^ Private Practice Tehran Iran

**Keywords:** artificial intelligence (AI), computerized tomography (CT), cone‐beam computerized tomography (CBCT), convolutional neural networks (CNN), deep learning, digital dentistry, machine learning

## Abstract

**Objectives:**

3D segmentation of the upper airway is crucial for dental and medical practices. However, it is a difficult and daunting task. Like almost all other areas, AI can theoretically help in airway segmentation. Nevertheless, AI's efficacy remains unknown. This meta‐analysis investigated this matter for the first time.

**Material and Methods:**

**‎**Various search engines/databases/articles were searched for articles published until April 25, 2025. All English‐language articles on the use of AI for upper airway evaluations based on CBCT or CT scans were included in the study. The desired population was considered studies assessing the upper airway. Intervention was the use of any tool of AI such as deep learning and machine learning for image analysis. The comparator was the manual analysis of CBCT or CT scans by human. The outcome was the analysis of upper airway on CBCT or CT images. The recorded and analyzed effect sizes were: accuracies, precisions, dice similarity scores, total volume differences, intersection over union (IoU), recall, or any other parameters relevant to segmentation. A meta‐analysis was conducted for each of the mentioned parameters if adequate data were available. The outcome was the analysis of upper airway on CBCT or CT images (PROSPERO: CRD42024508004).

**Results:**

Eleven studies were included, with 6 studies included in meta‐analyses. Most studies had a low risk of bias in most aspects. The qualitative part of review showed promising results for AI segmentation. Four of the effects sizes were meta‐analyzed: Precision,‎ dice similarity score, intersection over union, ‎ and recall were all above 90%.‎ Total volume difference was small but significantly above zero. Sensitivity analyses showed robustness of all meta‐analysis results. Publication bias was insignificant.

**Conclusions:**

The results showed promising AI efficacies in 3D segmentation of the upper airway in CBCTs. However, much more studies are needed before decisive conclusions.

## 1 Introduction

The analysis of upper airway anatomy plays a crucial role in the diagnosis and treatment planning of various respiratory disorders, including obstructive sleep apnea (OSA) and craniofacial anomalies (Orhan et al. 2022; Mupparapu et al. 2021). Cone‐beam computed tomography (CBCT) or computed tomography (CT) imaging has emerged as a valuable modality for capturing detailed three‐dimensional (3D) images of the upper airway (Neelapu et al. 2017; Sobouti et al. 2022). However, the manual interpretation of CBCT or CT scans for upper airway analysis can be time‐consuming, subjective, and prone to inter‐observer variability (Parks 2014; Rasteau et al. 2022).

To address these challenges, researchers have started exploring the potential of artificial intelligence (AI) techniques in automating the analysis of upper airway based on CBCT images. AI refers to the development of computer algorithms that can mimic human intelligence and decision‐making processes (Obermeyer and Emanuel 2016; Gharavi and Faghihimehr 2022). By leveraging machine learning algorithms, AI can extract meaningful information from large datasets and make accurate predictions based on learned patterns (Patil et al. 2022). AI has recently become very powerful and thus can be useful in medicine and ‎dentistry. It is a very novel and exciting area of research and practice full of cutting‐edge technological ‎advancements.‎

Recent studies have demonstrated promising results in the application of AI for upper airway analysis. For instance, researchers have developed AI models that can automatically segment the upper airway structures from CBCT images with high accuracy (Leonardi et al. 2021; Park et al. 2021). These models utilize convolutional neural networks (CNNs) and others to learn spatial features and classify different anatomical regions within the upper airway. The automated segmentation provided by AI algorithms not only saves time but also reduces the risk of human errors and inter‐observer variability (Cho et al. 2022; Shujaat et al. 2021; Alahmari et al. 2025).

Furthermore, AI techniques have been employed to quantify various upper airway parameters, such as the airway volume, its cross‐sectional area, and its shape irregularities (Leonardi et al. 2021; Park et al. 2021; Shujaat et al. 2021; de Bataille et al. 2023; Sin et al. 2021). By extracting these quantitative measures, AI algorithms can assist clinicians in assessing the severity of upper airway obstructions and predicting treatment outcomes. This objective analysis can provide valuable insights into patient‐specific treatment planning and monitoring (de Bataille et al. 2023; Sin et al. 2021).

Moreover, AI‐based approaches have shown potential in predicting OSA severity based on CBCT images. Researchers have developed predictive models that utilize AI algorithms to analyze upper airway morphology and identify anatomical features associated with OSA (Yun et al. 2019). These models can aid in the early detection and risk assessment of OSA, enabling timely interventions and personalized treatment strategies.

To date, no meta‐analysis has summarized studies that used AI to analyze the upper airway on CBCT or CT scans. Therefore, we aimed to systematically appraise such studies and possibly conduct meta‐analyses.

## 2 Materials and Methods

### 2.1 Information Sources and Search Strategy

The search was done for all available studies published until April 25, 2025. To find studies on the use of AI for upper airway evaluations based on CBCT or CT images, English‐language articles published until April 25, 2025, were searched by at least two authors, in the PubMed, Scopus, Web of Science, Cochrane Library, Institute of Electrical and Electronics Engineers (IEEE), and arXiv databases. Related terms were searched in the Medical Subject Headings (MeSH) database, and finally, the main search keywords were selected as follows: (“Artificial Intelligence” OR “Neural Network” OR “Deep Learning” OR “Machine Learning” OR “Robotics”) AND (“Apnea” OR “Airway”) AND “Computed Tomography.” Moreover, manual search was done in Google Scholar and also in reference lists of found full articles. The review was in accordance with PRISMA 2020; it was reviewed and approved by PROSPERO (CRD42024508004). The review protocol was not registered beforehand.

### 2.2 Inclusion and Exclusion Criteria

All English‐language articles on the use of AI for the evaluation of the upper airway (nasal and pharyngeal airways, from the ANS‐PNS line to the epiglottis) based on CBCT or CT volumes were included in the study. Regarding the population, intervention, comparison, and outcomes (PICO) criteria, the desired population was considered studies assessing the upper airway. Intervention was the use of any tool of AI such as deep learning and machine learning for image analysis. The comparator was the manual analysis of CBCT or CT images by human. The outcome was the analysis of the upper airway on CBCT or CT scans.

The following articles were excluded: reviews, case reports, editorials, guidelines, letter to the editors, and abstracts from conferences; articles not written in English; duplicate articles; articles with no available full text; articles which did not assess CBCT or CT images and considered other radiographic images such as Magnetic resonance imaging (MRI); and articles which did not assess upper airway, only evaluating lower airway space or merely comparing different skeletal malocclusions.

### 2.3 Types of Studies to be Included

All original studies will be included. Reviews, case reports, editorials, guidelines, letter to the editors, and abstracts from conferences will be excluded.

### 2.4 Condition or Domain Being Studied

CBCT or CT scans could be used to analyze different anatomic landmarks, including airway space. Several AI methods have been utilized in studies to analyze different parameters in the upper airway based on CBCT or CT images. The aim of this study was to systematically appraise studies that used AI in analyzing the upper airway on CBCT or CT images.

### 2.5 Population

The population comprised studies assessing the upper airway in CTs or CBCTs.

### 2.6 Intervention or Exposure

Use of any tool of AI such as deep learning and machine learning for CBCT or CT analysis.

### 2.7 Data Items

Almost all the contact authors (i.e., corresponding authors and any other authors with email addresses provided) of included studies were contacted and asked for their statistical raw data or at least their detailed information. None of the contacted authors responded to our emails.

The data were collected by at least two authors. To be included in the quantitative part (the meta‐analysis) regarding any method of AI, at least two different studies with that particular method needed to exist. The other variables for which data were sought were the authors’ names, year of publication, data modality, dataset size (training, validation, and testing), AI task, hardware, AI model structure, outcome, and mean (SD) for different outcomes (e.g., airway volume, precision of AI model, etc.).

### 2.8 Risk of Bias Assessment

The Cochrane Quality Assessment of the ACROBAT‐NRSI tool was used to evaluate the methodology of the included studies and determine whether there were any applicability or bias problems. Based on the following, each domain was evaluated and classified as high risk, low risk, or unclear:

1. Low risk of bias if all key domains of the study were at low risk of bias.
2. Unclear risk of bias if one or more key domains of the study were unclear.
3. High risk of bias if one or more key domains were at high risk of bias.

### 2.9 Synthesis of Results and Meta‐Analysis

The effect sizes were the following estimates: accuracies, precisions, dice similarity scores, total volume differences, intersection over union (IoU), recall, or any other parameters relevant to segmentation. A meta‐analysis was conducted for each of the mentioned parameters, if more than one article had reported adequate information about it. In most cases, studies did not report all the necessary information, therefore meta‐analyses could be conducted for four parameters: weighted means for dice similarity score, precision, recall, and total volume difference.

Sensitivity analysis was conducted through the leave‐one‐out meta‐analysis. Publication bias was estimated using the Egger regression; funnel plots were evaluated as well (if applicable). The software in use was STATA 17 (Stata, USA). The level of significance was set at 0.05.

## 3 Results

### 3.1 Study Selection

**Figure 1:**
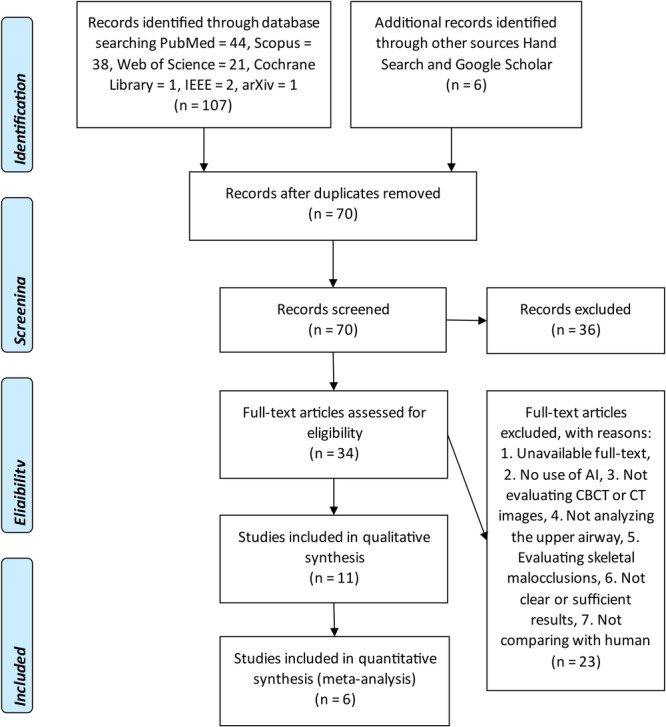
The flow diagram of studies included in this systematic review and meta‐analysis.

A total of 113 studies were found in search (Figure 1). Any duplicates were identified and removed, resulting in 70 unique articles. The abstracts of these 70 articles were screened. A total of 36 articles were marked as not relevant. The rest of articles (*n* = 34) were read and checked against the exclusion and inclusion criteria. A total of 23 articles were excluded because (Orhan et al. 2022) their full‐text was not available, (Mupparapu et al. 2021) AI was not used, (Neelapu et al. 2017) CBCT or CT was not used, (Sobouti et al. 2022) the upper airway was not assessed, (Parks 2014) skeletal malocclusions were evaluated, (Rasteau et al. 2022) they lacked the comparison with human gold standard, and (Obermeyer and Emanuel 2016) their results were not clear. Finally, 11 studies were included in the qualitative analyses and 6 of them were also eligible for meta‐analyses (Figure 1). Most of the studies showed low risks of bias in all measurements (Table 1). The summary of the included studies is given in Table 2.

**Table 1:** Risk of bias assessment.

Author, year (reference)	Bias due to confounding	Bias in the selection of participants into the study	Bias in the measurement of interventions	Bias due to departures from intended interventions	Bias due to missing data	Bias in measurement outcomes	Bias in the selection of the reported result	Overall bias
Alsufyani et al. (2016)	L	L	L	L	L	L	L	L
Leonardi et al. (2021)	L	L	L	L	L	B	L	B
Park et al. (2021)^a^	L	L	L	L	L	B	L	a
Shujaat et al. (2021)	L	L	L	L	L	L	L	L
Sin et al. (2021)	L	L	L	L	L	L	L	L
Orhan et al. (2022)	L	B	L	L	L	B	L	B
Cho et al. (2022)	L	L	L	L	L	L	L	L
Chu et al. (2023)	L	L	L	L	L	L	L	L
Jin et al. (2023)	L	L	L	L	L	L	L	L
Gao et al. (2024)	L	L	L	L	L	L	L	L
Süküt et al. (2025)	L	L	L	L	L	L	L	L

a The report by Park et al. (2021) had inconsistencies in terms of the reported sample size as well as the total volume difference. The items of this ‎table were all green from the report.‎ The sample size is once reported as 63 and once as 61. The total volume difference is once reported as ‎85.256 (‎‎86.504) and once as ‎‎137.256 (‎‎146.517). L, a low risk of bias; B, a high risk of bias.

**Table 2:** A summary of the included studies.

Author, year (reference)	Data	Dataset size (train/valid/test)	AI task	Hardware	Model structures	Outcome	Outcome mean (SD)	Comments
Alsufyani et al. (2016)	CBCT	10 case/10 control	Segmentation		Segura software (University of Alberta, Edmonton, Alberta, Canada)	Total volume difference (mm^3^)	1.9 (1.4)	They concluded the software is worthwhile in terms of precision and convenience.
						Total surface difference (mm^2^)	5.4 (3.6)	
						Time of segmentation‐human (s)	109 (9.4)	
						Time of segmentation‐Segura (s)	49 (11)	
Leonardi et al. (2021)	CBCT	20 training/20 test	Segmentation	Titan X Pascal GPU (NVIDIA Corporate, Santa Clara, Calif), Core i7 CPU with 8 GB RAM (Intel, Santa Clara, Calif)	CNN: Mimics software (version 20.0; Materialise, Leuven, Belgium)	Volume difference (cm^3^)	1.93 (0.73)	They found the AI segmentation accurate.
Park et al. (2021)	CBCT	252 training/63 test or 61 test?	Segmentation		Regression Neural Network‐based deep‐learning: MATLAB 2020a (MathWorks, Natick, MA, USA)	Total volume (mm^3^)	85.256 (86.504) or 137.256 (146.517)	The AI method was appropriate. There were some inconsistencies
Shujaat et al. (2021)	CT/CBCT	48 train/30 validation/25 test	Segmentation		3D U‐Nets CNN: Mimics software (version 22.0, materialize N.V., Leuven, Belgium).	Accuracy (%)	1 (0)	The AI algorithm was accurate.
						Precision (%)	0.97 (0.01)	
						Recall (%)	0.96 (0.03)	
						Dice score (%)	0.97 (0.02)	
						IoU (%)	0.93 (0.03)	
Sin et al. (2021)	CBCT	214 training/46 validation/46 test	Segmentation	NVIDIA GeForce RTX 2080 Ti GPU	U‐Net: Open‐source version 3.8 ITK‐SNAP software	Accuracy (%)	0.961	The accuracy of AI software was confirmed.
						Dice score (%)	0.919	
						IoU (%)	0.993	
						Volume‐human (cm^3^)	18.08 (0.52)	
						Volume‐AI (cm^3^)	17.32 (0.50)	
Orhan et al. (2022)	CBCT	100 OSA and 100 non OSA (180 training/10 validation/10 test)	Segmentation	NVIDIA GeForce RTX A100 GPU	Human/Diagnocat, InVivo 5/Developed AI	Narrowest points of the airway (mm) – OSA	6.31/6.10/NA	Both AI methods were accurate.
						Narrowest points of the airway (mm) – non OSA	5.96/5.70/NA	
						The field of the airway (mm^2^) – OSA	1057.59/1013.90/NA	
						The field of the airway (mm^2^) – non OSA	883.41/930.02/NA	
						Volume of the airway (cm^3^) – OSA	19.63/20.25/18.27	
						Volume of the airway (cm^3^) – non OSA	17.95/18.50/17.96	
Cho et al. (2022)	CBCT	100 training/16 validation/100 test	Segmentation	Intel Xeon E5‐2620 v4 CPU at 2.10 GHz and 4 Nvidia Titan RTX GPUs, and 256 GB of RAM	3D U‐Net‐based CNN: Mimics software (version 20.0; Materialise, Leuven, Belgium)	Precision (%)	0.925 (0.030)	The accuracy of the AI software was high.
						Recall (%)	0.921 (0.029)	
						Dice score (%)	0.928 (0.023)	
						Volumetric similarity	0.951 (0.022)	
Chu et al. (2023)	CBCT	161 training/161 validation/40 test	Segmentation and localization	PC with an Intel i7‐8700 CPU, 32GB RAM and a single Nvidia RTX 2080 Ti GPU with 12 G VRAM	Mimics v19.0 (Materialise, Leuven, Belgium): U‐Net18/U‐Net36/DeepLab50/DeepLab101	Precision (%)	90.2	The accuracy and efficacy of the AI models were appropriate.
							90.1	
							90.0	
							90.6	
						Recall (%)	89.2	
							88.9	
							88.0	
							88.6	
						Dice score (%)	81.2	
							80.9	
							79.8	
							80.9	
						IoU (%)	89.4	
							89.1	
							88.4	
							89.2	
						Minimal cross‐sectional area (mm^2^)	206 (123)	
						Lateral dimension of the minimal cross‐sectional area (mm)	27 (7)	
						Anteroposterior dimension of the minimal cross‐sectional area (mm)	10.2 (3.6)	
Jin et al. (2023)	CBCT	32 training/8 validation/10 test	Segmentation	12 GB NVIDIA RTX 3060 GPU	Swin Transformer and U‐Net based CNN: Vision Transformer	Precision (%)	94.25 (1.72)	A high AI precision was achieved.
						Recall (%)	98.44 (1.42)	
						Dice score (%)	96.29 (0.94)	
						IoU (%)	92.85 (1.73)	
		15			Human/Vision Transformer	Total upper airway volume (mm^3^)	46051.52 (10731.33)/46109.93 (10749.33)	
		15			Vision Transformer/Dolphin software (version 11.95; Dolphin Imaging and Management Solutions, Chatsworth, Calif, USA)	Total upper airway volume (mm^3^)	30604.63 (10239.25)/30562.47 (10184.91)	
Gao et al. (2024)	CBCT	Study main dataset: 950 training/238 validation/157 test	Segmentation	16 GB NVIDIA GeForce RTX 3080 Ti GPU	U‐Net/Atten‐Unet/ResUnet/Unet + +/DeepLabv3 + /DCSAU_Net/TransUnet/RELA_Net	Precision (%)	98.70/98.42/98.22/98.74/98.30/98.26/98.06/98.31	Their RELA_Net outperformed many other models in terms of Dice score and IoU.
						Recall (%)	94.51/95.36/94.63/93.77/95.11/95.56/95.80/95.97	
						Dice score (%)	96.46/96.83/96.32/96.14/96.63/96.85/96.88/97.10	
						IoU (%)	93.32/93.90/93.00/92.66/93.53/93.96/94.01/94.39	
		DSB 2018 dataset: 428 training/107 validation/135 test			U‐Net/Atten‐Unet/ResUnet/Unet + +/DeepLabv3 + /DCSAU_Net/TransUnet/RELA_Net	Precision (%)	91.02/91.09/88.39/89.77/88.27/89.63/90.93/91.21	
						Recall (%)	92.17/92.56/91.62/93.38/89.33/92.15/92.85/92.63	
						Dice score (%)	91.03/91.40/89.51/91.10/88.27/90.11/91.24/91.50	
						IoU (%)	84.26/84.79/82.66/84.20/80.53/83.11/84.55/84.96	
Süküt et al. (2025)	CBCT	60 training/10 validation/30 test	Segmentation		MONAI Label: CNN‐based model/semi‐automatic ITK‐SNAP software (version 4.0.2, www.itksnap.org)	Precision (%)	97.1 (1.7)/96.2 (2.4)	AI results resembled manual results.
						Recall (%)	87.8 (4.8)/92.1 (3.9)	
						Dice score (%)	91.5 (4.1)/94.0 (2.1)	
						95% Hausdorff distance (mm)	1.45 (0.67)/0.98 (0.58)	

#### 3.1.1 Meta‐Analysis

Meta‐analyses were performed whenever there were two or more studies with adequate information in each category.

##### 3.1.1.1 Outcome 1: Precision

**Figure 2:**
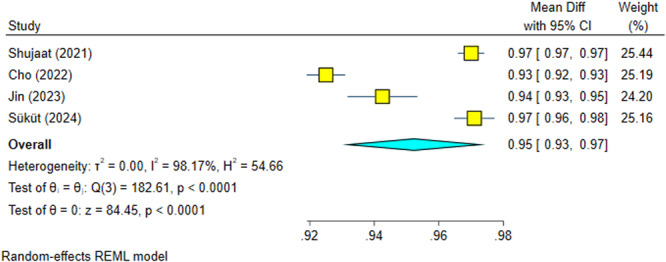
Weighted mean differences (and 95% CIs) for the precision of the AI systems.

**Figure 3:**
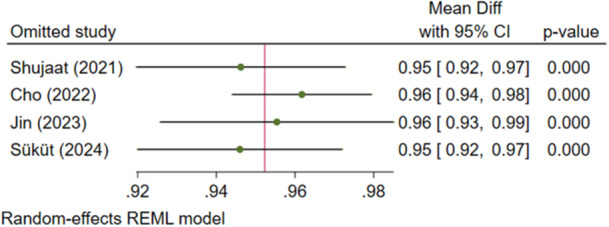
Sensitivity analysis for the precision of the AI systems.

This meta‐analysis included four articles (Cho et al. 2022; Shujaat et al. 2021; Jin et al. 2023; Süküt et al. 2024) and had a high heterogeneity (*I* ^2^ = 98.17%, *p* = 0.0000). The overall effect size was 95% and significantly high, with 95% confidence intervals above 90%, indicating very high precisions (Figure 2). Sensitivity analysis confirmed the robustness of the findings (Figure 3). The Egger regression showed no significant publication bias (*p* = 0.535).

##### 3.1.1.2 Outcome 2: Dice Similarity Score

**Figure 4:**
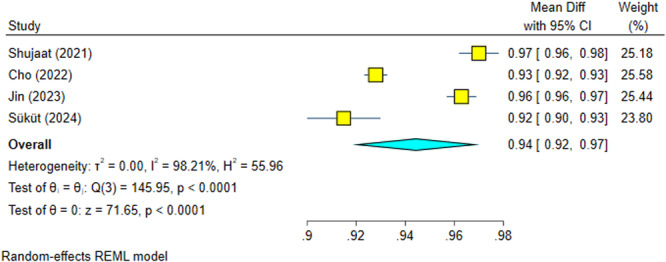
Weighted mean differences (and 95% CIs) for the dice score of the AI systems.

**Figure 5:**
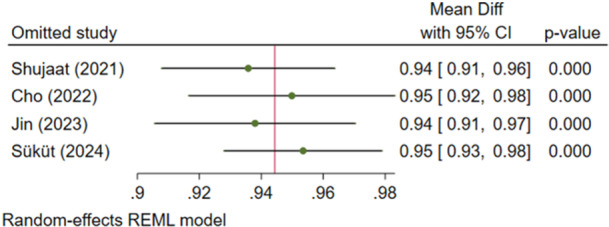
Sensitivity analysis for the dice score of the AI systems.

This meta‐analysis had four reports (Cho et al. 2022; Shujaat et al. 2021; Jin et al. 2023; Süküt et al. 2024) with significant heterogeneity (*I* ^2^ = 98.21%, *p* = 0.0000). The overall effect size was 94.4% and significantly high, with 95% confidence intervals resting above 90% dice similarity (Figure 4). Sensitivity analysis confirmed the robustness of the findings (Figure 5). The Egger regression detected no significant publication bias (*p* = 0.425).

##### 3.1.1.3 Outcome 3: Total Volume Difference

**Figure 6:**
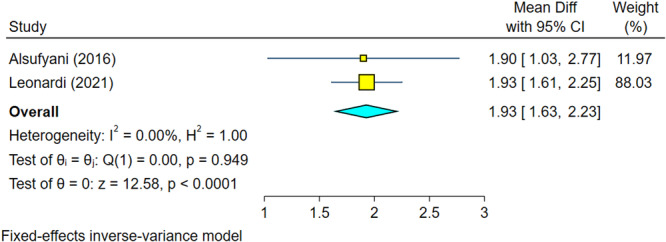
Weighted mean differences (and 95% CIs) for the total volume difference of the AI.

**Figure 7:**
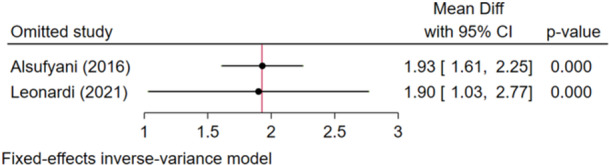
Sensitivity analysis for the total volume difference of the AI.

Two studies without heterogeneity were analyzed (*I* ^2^ = 0.0%, *p* = 0.949) (Leonardi et al. 2021; Alsufyani et al. 2016). According to the overall effect size, the total volume difference of AI systems used in these studies was 1.93 and significantly different from zero, indicating that volumes calculated by AI differ from those measured by human (Figure 6). However, the overall difference was clinically small. Sensitivity analysis confirmed the robustness of the findings (Figure 7). The Egger regression showed no publication bias (*p* = 0.949).

##### 3.1.1.4 Outcome 4: Recall (Sensitivity)

**Figure 8:**
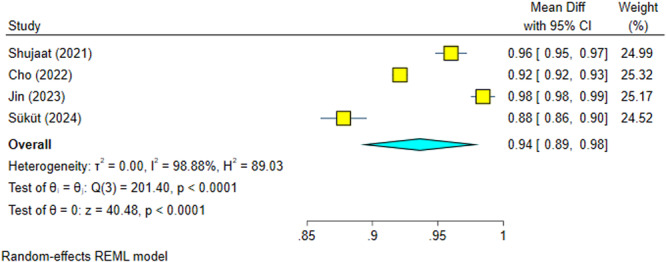
Weighted mean differences (and 95% CIs) for the recall of the AI systems.

**Figure 9:**
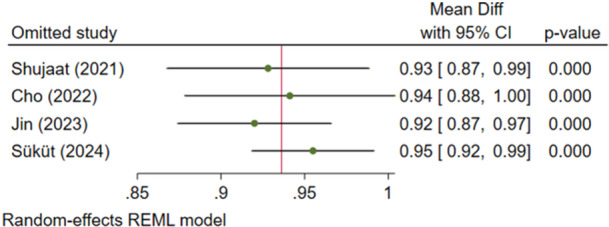
Sensitivity analysis for the recall of the AI systems.

Four studies with significant heterogeneity (*I* ^2^ = 98.88%, *p* = 0.0000) were included (Cho et al. 2022; Shujaat et al. 2021; Jin et al. 2023; Süküt et al. 2024). The recall (sensitivity) of AI systems used in these studies was significantly high (94%), with 95% confidence intervals almost above 90% (Figure 8). Sensitivity analysis confirmed the robustness of the findings (Figure 9). The Egger regression did not show a significant publication bias (*p* = 0.377).

##### 3.1.1.5 Outcome 5: Intersection Over Union (IoU)

**Figure 10:**
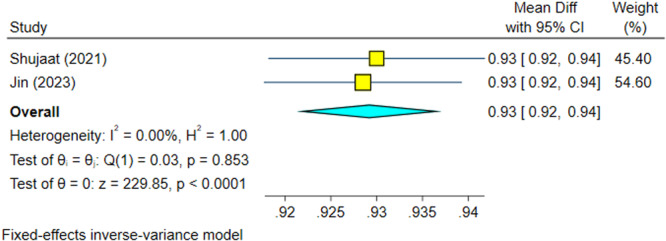
Weighted mean differences (and 95% CIs) for the intersection over union of the AI systems.

**Figure 11:**
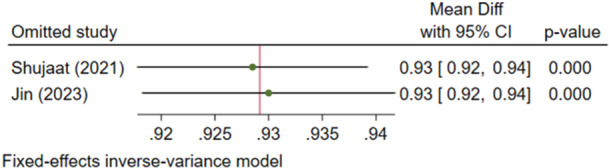
Sensitivity analysis for the intersection over union of the AI systems.

Two studies (Shujaat et al. 2021; Jin et al. 2023) examined IoU, with no significant heterogeneity (*I* ^2^ = 0.00%, *p* = 0.853). The IoU of AI systems was significantly high (92.9%), with 95% confidence intervals above 90% (Figure 10). Sensitivity analysis confirmed the robustness of the findings (Figure 11). The Egger regression did not show a significant publication bias (*p* = 0.853).

## 4 Discussion

The meta‐analyses showed very high precision, dice score, and recall. It showed that there is a small but significant difference between the upper airway volume computed via AI versus human.

An essential image processing stage in the study of medical images is the segmentation of 3D images. High reproducibility and accuracy and low bias are important objectives in medical imaging (Taha and Hanbury 2015). Properly identifying the patterns of change is crucial for timely illness diagnosis and effective disease surveillance. As a result, evaluating the quality and accuracy of segmentation algorithms is crucial (Taha and Hanbury 2015). Examples of anatomic features are white matter, gray matter, lesions of the brain, body organs and tumors. Segmentation evaluation is the task of comparing two segmentations by measuring the distance or similarity between them, where one is the segmentation to be evaluated and the other is the corresponding ground truth segmentation (Taha and Hanbury 2015). Depending on the body components being imaged and the image resolution, medical 3D scans are specified on a 3D grid that can have varying sizes. The width, height, and depth of the 3D scan are indicated by the grid size, which is expressed as (w×h×d). A voxel is any 3D point on the grid. A binary segmentation may be thought of as a partition that, given an anatomic characteristic, categorizes the voxels of an image based on whether or not they belong to a feature (Taha and Hanbury 2015). Medical segmentation is usually done using fuzzy logic. In this context, segmentations may be seen as the likelihood that voxels will belong to specific classes. Thresholding the probabilities at a certain value yields binary representations that may be assessed as crisp segmentations, which is one method of analyzing fuzzy segmentations. Nevertheless, thresholding is only an imperfect solution that offers a rough approximation (Taha and Hanbury 2015). Besides, defuzzifying needs selecting a threshold, which is again challenging and crucial, since diagnosis depends on the threshold (Taha and Hanbury 2015). The quality of medical 3D image segmentation is evaluated using various metrics (Taha and Hanbury 2015). For instance, Fenster and Chiu (2005) considered the following metrics as important segmentation assessments: 1. Accuracy, which is the extent of agreement between the segmentation output and the segmentation done by the expert human as the gold standard. This metric can have 3 aspects: size or volume of the segmented item, the contour or boundary of the segmented object, and the object's alignment, that is, its general position which may become more important for small items. 2. Precision, which indicates reproducibility. 3. Efficiency which is about time (Taha and Hanbury 2015; Fenster and Chiu 2005). Recall or sensitivity, also known as true positive rate, concerns the extent of positive voxels in the gold standard (ground truth) which are also detected as positive by the segmentation algorithm (Taha and Hanbury 2015). It is calculated as the extent of true positive divided by the sum of true positive and false negative. It should be noted that this measure is sensitive to the size of the area to be segmented in a way that errors in small areas would become more highlighted than errors in larger segments (Taha and Hanbury 2015; Fenster and Chiu 2005; Gerig et al. 2001; Udupa et al. 2006). Precision concerns the extent of true positive results divided by the sum of true positive and false positive (Taha and Hanbury 2015). Accuracy concerns the extent of how much the result is close to the ground truth. Also called pixel accuracy or Rand index, it is a very famous metric in statistical assessments (Taha and Hanbury 2015; Müller et al. 2022). It is defined as correct negative and positive predictions in comparison with all predictions, or as the sum of true positives and true negatives divided by the sum of true positive, true negative, false positive, and false negative. It should be noted that accuracy is likely to be inflated for reasons such as the inclusion of true negatives (Müller et al. 2022). Some of the metrics concern with the overlap between the ground truth and AI segmentation. Metrics based on F‐score or F‐measure are very common in AI image processing (Taha and Hanbury 2015; Müller et al. 2022; Popovic et al. 2007). F‐score is computed from the precision and sensitivity, indicating the overlap between the ground truth and predicted segmentation. It is appropriate for medical imaging segmentation since it penalizes false positives which are frequent in medical images (Taha and Hanbury 2015; Müller et al. 2022; Popovic et al. 2007). Metrics that use F‐measure include dice similarity coefficient (dice score) and the Intersection‐over‐Union (IoU) (Taha and Hanbury 2015; Müller et al. 2022). Dice score or dice coefficient also known as the overlap index, is a very usually used metric in 3D segmentations, which may indicate reproducibility as well as an automatic‐manual comparison (Taha and Hanbury 2015). It is calculated as 2 × true positive divided by the sum of false negative, false positive, and 2 × true positive (Taha and Hanbury 2015). The intersection over union has a rather similar definition, except that it penalizes more severely over‐segmentations and under‐segmentations (Müller et al. 2022). It is calculated as true positive divided by the sum of false negative, false positive, and true positive (Taha and Hanbury 2015; Müller et al. 2022; Popovic et al. 2007). The studies reviewed by us showed high extents of various metrics implying relative success of AI in segmentation tasks. The meta‐analyses were all statistically significant; when it comes to recall, dice score, and precision, the very high overall means indicated satisfactory efficacies of the AI. Still, the airway volumes calculated by the AI was slightly but significantly larger than that calculated by humans. While the mean difference of 1.93 cm^3^ appears statistically significant, its clinical implication warrants nuanced interpretation. In the context of diagnosing OSA, airway volume is a critical parameter; however, systematic reviews and reliability studies indicate that human inter‐observer variability for upper airway segmentation often exceeds these, due to subjective boundary definitions and threshold selection (Zimmerman et al. 2019, 2016). Consequently, the error margin exhibited by AI falls within the range of typical human disagreement. Nevertheless, for precise treatment planning, such as the fabrication of Mandibular Advancement Devices (MAD), systematic overestimation by AI could theoretically influence the projected efficacy of an intervention. For instance, Haskell et al. reported that MAD therapy typically increases oropharyngeal volume by approximately 2.8 cm^3^ (Haskell et al. 2009). Given that the AI measurement error (1.93 cm^3^) represents nearly 70% of this therapeutic effect size, clinicians should remain cautious when relying solely on automated volumetric data for borderline OSA cases where minor volumetric changes dictate treatment modality. Apart from the volume differences, other metrics showed high heterogeneities among the studies, which point to high variations in results. This high heterogeneity likely stems from substantial methodological variability across the primary studies. Specifically, inconsistencies in the definition of anatomical boundaries may introduce noise into the ground truth data used to train these models (Zimmerman et al. 2019, 2016). Furthermore, the variation in imaging acquisition parameters (e.g., voxel size, field of view, and artifacts from different CBCT scanners) creates a “domain shift” where an algorithm trained on scans from one vendor may fail to generalize to those from another (Zimmerman et al. 2019, 2016; Liang et al. 2020).

While some studies used CT, most used CBCT. CT scans are frequently utilized for more comprehensive craniofacial evaluations, which may affect segmentation accuracy, even if CBCT offers high‐resolution imaging for oral structures. Disparities in reported measures were probably caused by variations in contrast settings, voxel sizes, and imaging techniques (Alahmari et al. 2025). The lack of a standardized segmentation protocol across the included studies prevents a direct “apples‐to‐apples” comparison of algorithmic efficiency.

This systematic review was limited by some factors. The number of studies meeting the inclusion criteria for qualitative synthesis was small. Crucially, the generalizability of the reported findings is constrained by the prevalence of small, single‐center datasets in the included studies. Deep learning models trained on limited cohorts are prone to overfitting, capturing the specific noise patterns or demographic characteristics of a single institution rather than learning robust anatomical features applicable to a diverse global population. Additionally, the reliance on proprietary, closed‐source pipelines in several studies hinders reproducibility and external validation. Without access to the source code or the specific train‐test splits, independent researchers cannot verify the reported high accuracy rates, raising concerns about potential “black box” bias where models perform well only on their internal validation sets (Haibe‐Kains et al. 2020). Additionally, when contacted, all authors of all earlier studies did not respond. The non‐response of those authors to our data requests might introduce a small possibility of selective reporting bias, where less favorable results might have not been reported. Of course, there may be other explanations as well; for instance, all those authors might have missed our data request emails. More importantly, many of them could not be entered into quantitative meta‐analyses because their authors had reported insufficient information and refused to reply to our email asking for data or more information. Therefore, the outcome of the performed four meta‐analyses should be approached cautiously. These limitations lower the certainty and conclusiveness of the results. Future research should move beyond simple efficacy metrics and focus on robustness and standardization. To overcome the current fragmentation, the development of large‐scale, multicenter open‐access datasets is imperative to benchmark algorithms transparently. Future studies should also adhere to reporting guidelines to ensure that essential details regarding data partitioning, model architecture, and failure analysis are explicitly documented.

## 5 Conclusion

In conclusion, the evaluated 11 studies unanimously indicated that AI techniques for upper airway segmentation of CBCT or volumes might be of value. The meta‐analyses showed very high precision, dice score, and recall. There might be a small but significant difference between the upper airway volume computed via AI versus human. Nevertheless, these conclusions are not decisive, since the number of studies with full information was a few and future studies are warranted in this regard.

## Author Contributions

Farhad Sobouti conceived and designed the study, searched the literature and collected the data, interpreted the findings, and contributed to the article. Mehdi Aryana searched the literature and collected the data, assessed the risk of bias, prepared the tables, interpreted the findings, and contributed to the article. Sepideh Dadgar conceived the study, searched the literature and collected the data, interpreted the findings, and contributed to the article. Reza Alizadeh Navaei designed the study, contributed to statistical analyses, and interpreted the findings. Hossein Mohammad‐Rahimi contributed to search, data extraction, and interpretations. Vahid Rakhshan checked and validated the meta‐data, contributed to data extraction, contacted the authors for more data or details, contributed to risk‐of‐bias assessments and tables, performed statistical analyses, prepared the figures, interpreted the findings, and wrote and revised the article. All the authors approved the final version and agreed to submit to this journal.

## Funding

The authors received no specific funding for this work.

## Ethics Statement

This review did not include any experiments on any human subjects or animals or any other experiments. The review was in accordance with PRISMA 2020; it was reviewed and approved by PROSPERO (CRD42024508004).

## Consent

The authors have nothing to report.

## Conflicts of Interest

The authors declare no conflicts of interest.

## Transparency Statement

All authors affirm that this manuscript is an honest, accurate, and transparent account of the study being reported; that no important aspects of the study have been omitted; and that any discrepancies from the study as planned (and, if relevant, registered) have been explained. No generative AI tool was used for writing this paper. However, AI was used to improve the grammar and fluency of some sentences in the discussion. All authors have read and approved the final version of the manuscript. All authors had full access to all of the data in this study and take complete responsibility for the integrity of the data and the accuracy of the data analysis.

## Data Availability

The data are already presented as Table 1 and forest plots.
